# High Sensitivity Detection of CdSe/ZnS Quantum Dot-Labeled DNA Based on N-type Porous Silicon Microcavities

**DOI:** 10.3390/s17010080

**Published:** 2017-01-01

**Authors:** Changwu Lv, Zhenhong Jia, Jie Lv, Hongyan Zhang, Yanyu Li

**Affiliations:** 1 School of Physical Science and Technology, Xinjiang University, Urumqi 830046, China; lvchw@xju.edu.cn (C.L.); zhy@xju.edu.cn (H.Z.); knockout_2003@163.com (Y.L.); 2 College of Information Science and Engineering, Xinjiang University, Urumqi 830046, China; 3 College of Resource and Environment sciences, Xinjiang University, Urumqi 830046, China; lvjie@xju.edu.cn

**Keywords:** N-type porous silicon, quantum dot labeling, QD-DNA, reflectance spectrum

## Abstract

N-type macroporous silicon microcavity structures were prepared using electrochemical etching in an HF solution in the absence of light and oxidants. The CdSe/ZnS water-soluble quantum dot-labeled DNA target molecules were detected by monitoring the microcavity reflectance spectrum, which was characterized by the reflectance spectrum defect state position shift resulting from changes to the structures’ refractive index. Quantum dots with a high refractive index and DNA coupling can improve the detection sensitivity by amplifying the optical response signals of the target DNA. The experimental results show that DNA combined with a quantum dot can improve the sensitivity of DNA detection by more than five times.

## 1. Introduction

It is advantageous to use porous silicon because of its simple preparation processes; the pore size, pore density, and porous silicon layer thickness can be controlled by changing electrochemical corrosion conditions (e.g., the silicon wafer type, the doping, the electrolyte ratio, and the corrosion current density) [1,2,3]. A large specific surface area can adsorb large amounts of chemical and biological molecules. These properties make porous silicon a good candidate for highly sensitive, unlabeled optical biochemical sensors [4,5]. The combination of a biomolecules and porous silicon can result in change of an effective refractive index. By observing the effect of this change on the reflectance spectrum shift such as a monolayer porous silicon interference peak shift [6,7], the Bragg reflector center position [8], or the microcavity defect state [9,10]—it is possible to detect biochemical molecules.

Quantum dots (QDs) are mostly used as high-sensitivity fluorescent molecule labels because of their high luminescence quality, good monochromaticity, surface functionalization, and tunable photoluminescence range [11,12]. Nanoparticles such as Au or QDs were attached to the DNA before the DNA was hybridized with the complementary DNA for optical sensing [13,14]. Many optical methods are used to observe the changes caused by the nanoparticle addition to the DNA such as light scattering, SPR, fluorescence, and SERS [15]. Moreover, the high refractive index of QDs can increase the effective refractive index of QD-labeled molecules while making them luminescent. This causes a greater reflectance spectrum shift, improving the sensitivity of the sensor. Girija Gaur et al. utilized reflectance spectrum shifts to detect QD-labeled small molecules of biotin in a porous silicon monolayer [16]. The result was a six-fold increase in detection sensitivity compared to that of unlabeled targets. We know that a porous silicon microcavity (PSM) is more sensitive to an effective refractive index change due to a sharp dip in the reflectance spectrum for the defect state. In addition, the pore size is an important factor that we must consider in sensing. The pore size of P-type porous silicon ranges from 10 nm to 30 nm [17], the size of the QD is about 4 nm, and the size of the 20-base DNA molecule is about 6 nm in length. Note that the size of the DNA molecule will increase after quantum dot labeling. A small ratio between pore size and molecule size will cause the QD-labeled DNA to plug the pore and prevent other biological molecules from entering the microcavity [18]. In comparison, N-type silicon pores are generally much larger (50 nm–120 nm) [19,20].

In this paper, N-type porous silicon microcavity structures are used. Hydroxyl-modified CdSe/ZnS water-soluble QDs are coupled with DNA molecules; the QDs are used to amplify the effective refractive index change.

**Figure 1:**
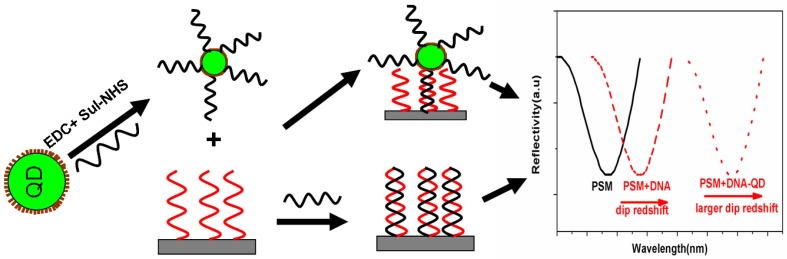
Schematic diagram of the sensing principle.

The schematic image of the sensing principle is shown in Figure 1. After the hybridization between the complementary DNA and the probe DNA in the PSM structure, the effective refractive index of the structure changed, and the red shift of the spectrum can be observed in the reflection spectrum. QDs are marked by a series of chemical couplings to the target DNA, the effective refractive index of DNA-QD significantly increased due to the high refractive index of the quantum dots. The red shift in the reflectance spectrum increased compared to the unmodified DNA. QDs play a role in increasing the spectral response signal. This method increases the sensitivity of detecting DNA hybridization.

## 2. Experimental

### 2.1. Materials and Instruments

Ethanolamine hydrochloride (EA), 2-[4-(2-hydroxyethyl)-1-piperazinyl] ethanesulfonic acid (HEPES) buffer (pH 9.0), glutaraldehyde (GA, 50%), and aminopropyl triethoxysilane (APTES, 99%) were purchased from Shanghai Aladdin Reagent Co., Ltd. (Shanghai, China), CdSe/ZnS carboxyl-modified water-soluble quantum dots were purchased from Wuhan Jiayuan Quantum Dots Biotech Co., Ltd. (Wuhan, China), along with a phosphate buffer (PBS, pH 7.4), 1-(3-dimethylaminopropyl)-3-ethyl carbodiimide hydrochloride (EDC), and N-hydroxysulfosuccinimide (Sul-NHS). All purchased reagents were analytical grade.

Target DNA: 5′-CGCGGCCTATCAGCTTGTTG-3′-NH2,
Probe DNA: 5′-CAACAAGCTGATAGGCCGCG-3′-NH2.
DNA primers were purchased from INVITROGEN TRADING Co., Ltd. (Shanghai, China).

Field emission scanning electron microscopy (FESEM, ZEISS SUPRA 55VP) was used to characterize the porous silicon surface and cross section. The transmission electron microscopy (TEM) image was acquired using a 200 kV field emission transmission electron microscope (JEM-2100F). The absorption spectrum is measured using a Nano Drop 2000 UV-Vis spectrophotometer (Thermo Scientific, Waltham, MA, USA). The fluorescence emission spectrum was measured using a Hitachi UV-Vis spectrofluorometer (Hitachi F-4600, Tokyo, Japan). A Hitachi UV-Vis spectrophotometer (Hitachi U-4100, Tokyo, Japan) was used to measure the sample reflectance spectrum.

### 2.2. Porous Silicon Microcavity Preparation

**Figure 2:**
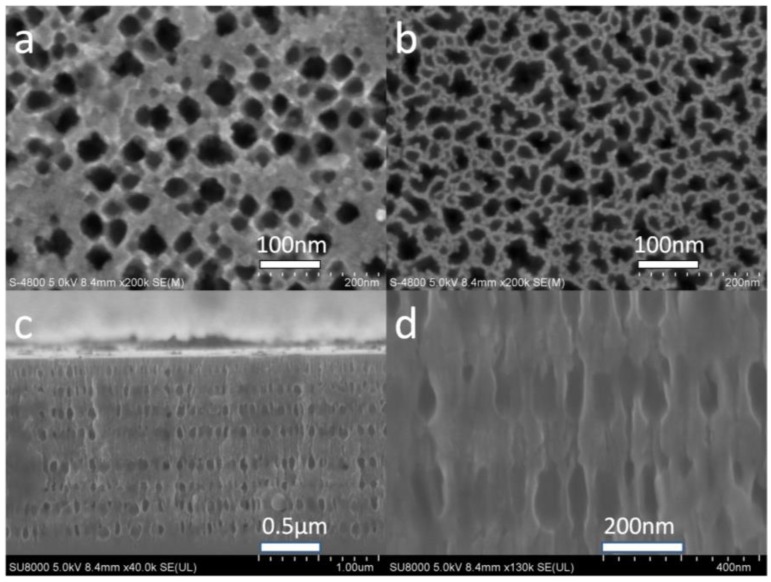
Scanning electron microscopy image (**a**) subjected to a corrosion current 14 mA and (**b**) subjected to a corrosion current 28 mA; (**c**) cross-sectional view of the PSM; (**d**) an enlarged view of the cross section.

N-type heavily doped silicon wafers with 0.01 ohm·cm to 0.02 ohm·cm and (100) crystal orientation were diced into 1 cm^2^ pieces. These pieces were subsequently cleaned using acetone, ethanol, and deionized water for 10 min each, and then dried in air. The samples were etched with a single cell under constant current, using a mixture of hydrofluoric acid and water; the HF acid concentration was roughly 5% (25 mL 40% HF: 200 mL water). The corrosion current density cycle was 14 mA for 4.5 s and 28 mA for 4 s, with an interval of 5 s. The defect layer was etched using a current density of 14 mA for 9 s. Note that the N-type silicon pore size is relatively large; the pores etched using corrosion currents of 14 mA and 28 mA are shown in Figure 2a,b, respectively. The corresponding pore sizes are roughly 30–40 nm and 40–50 nm. The pore diameters are large enough for the biomolecules and the quantum dots to enter the porous silicon. The pores are round, with relatively thick walls, as shown in Figure 2a. In Figure 2b, the pore shapes are irregular with thin walls. Figure 2c shows a cross-sectional view of the PSM. The defect layer is in the middle, with eight upper and eight lower layers. Figure 2d shows an enlarged view of the cross section.

### 2.3. Quantum Dot Coupled with DNA

**Figure 3:**
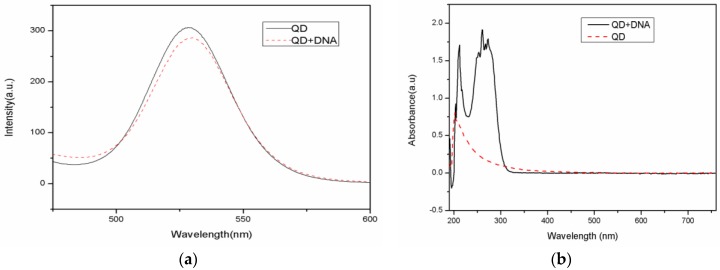
Fluorescence and absorption spectra of QD and the QD-labeled DNA: QD represents the fluorescence spectrum of the quantum dots, QD + DNA represents the fluorescence spectrum of the quantum dot-labeled DNA. (**a**) fluorescence spectra; (**b**) absorption spectra.

A 50 uL CdSe/ZnS carboxyl-modified water-soluble quantum dot solution (8 µM concentration) was diluted with PBS to 1 µM. Next, 40 µL of EDC (0.01 M concentration) and 40 µL of Sul-NHS (0.01 M concentration) were added to the reaction mixture. After reacting for 10 min, 50 µL of amino-modified DNA (40 µM concentration) was added and the reaction vessel was shaken gently for 10 h in the dark at room temperature. Centrifugation can remove excess DNA due to the solubility differences between DNA and carboxyl moiety on the QD. The QD-modified DNA separates because many unlinked carboxyl moieties on the surface of QD have less solubility than DNA. The QD-DNA weight is larger than the DNA, while the unlinked DNA is still in the solution. After centrifugation at 10,000 rpm for 10 min, the supernatant was removed and the precipitate was diluted with PBS into a different concentration of DNA solution and stored at 4 °C. The fluorescence emission spectra of the quantum dot and the quantum dot-DNA solution were measured from a 100 µL sample using an excitation wavelength of 370 nm, an excitation voltage of 400 v, and a slit width of 10 nm. The fluorescence and absorption spectra of QDs before and after DNA coupling are shown in Figure 3. The emission peak of carboxyl-modified CdSe/ZnS water-soluble quantum dots is located at 528 nm. Note that the peak shifted to 530 nm after the quantum dot coupled with DNA. The absorption spectrum shows that there is an added 260 nm absorption peak after the QD combined with DNA. Thus, both the fluorescence and absorption spectra indicate that the quantum dot and DNA conjunction was successful. Quantum dot-labeled DNA has little effect on the overall photoluminescence intensity.

### 2.4. PSM Functionalization

To immobilize the target biological molecules to the porous silicon surface, the PSM requires a series of functional processes to stabilize the porous silicon surface and modify the pore surface functional groups. First, the freshly-prepared PSM was oxidized; the samples were heated to 500 °C for 30 min in an oxidizing furnace. Next, they were soaked in 5% APTES for 1 h, rinsed with deionized water, dried in air, and incubated at 100 °C for 10 min. After silanization, the amino groups were immobilized to the pore surface. The samples were immersed in a 2.5% glutaraldehyde solution for 1 h, then rinsed with PBS (pH 7.4) and dried in air to modify the surface of the porous silicon with an aldehyde group. The aldehyde group can be coupled with amino-modified DNA.

### 2.5. DNA Probe Connected to Porous Silicon Microcavity

A 50 µL DNA probe (10 µM concentration) was dropped into the functionalized PSM sample using a pipette. After 2 h at 37 °C, the samples were rinsed with PBS to remove excess DNA. The excess/unreacted aldehyde functional groups were then closed with EA (3M in HEPES buffer, pH 9.0) incubated at 37 °C for 1 h. Finally, the samples were rinsed with PBS and dried in air.

### 2.6. Target DNA Detection

Fifty microliters of target DNA labeled with QDs at different concentrations were attached to the PSM samples. The samples were incubated at 37 °C for 2 h to allow DNA to hybridize. Afterwards, the samples were rinsed with PBS to wash away the unconnected target DNA and QDs, and then dried in air.

The sample reflectance spectra were measured after each step of sample functionalization (oxidization, silanization, GA) after probe combination and the hybridization of the DNA probe with QD-DNA.

## 3. Results and Discussion

**Figure 4:**
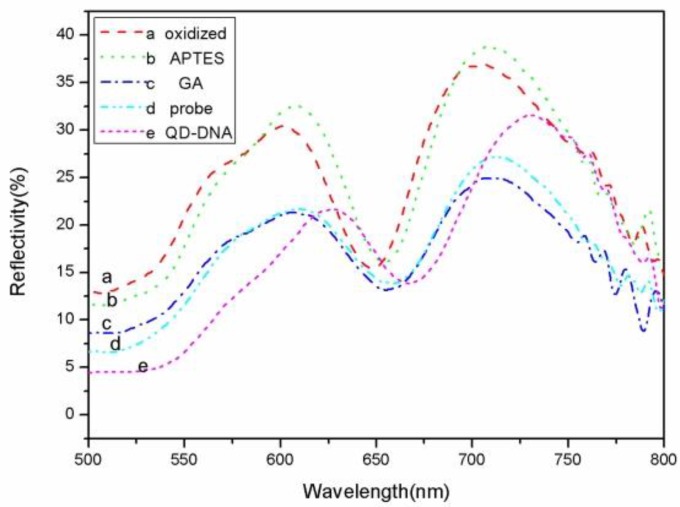
PSM reflectance spectrum following (**a**) oxidation; (**b**) silanization; (**c**) glutaraldehyde; (**d**) DNA probe; (**e**) QD-DNA target.

**Figure 5:**
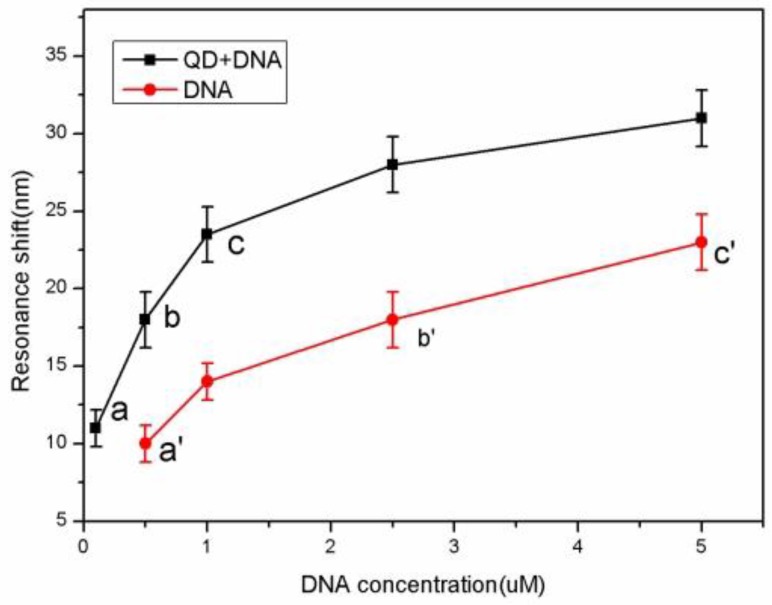
Relationship between the target DNA at different concentrations and the reflectance spectrum shift. The black squares represent the QD-DNA and the red dots represent the control DNA (unlabeled).

The defect state in the high reflection region of the PSM reflectance spectrum is near 650 nm. When the sample was oxidized, silanized, and functionalized with glutaraldehyde, and the probe DNA was hybridized with complementary target QD-DNA, the reflectance spectrum of the PSM showed a regular red shift. Figure 4 shows the reflectance spectrum shifts. The reflectance spectrum shift reflects the PSM refractive index changes that result from the functionalization and DNA connection. When a small molecule was grafted to the porous silicon surface, the effective refractive index of the porous silicon becomes large, and the reflectance spectrum shifts to the red. The amount of red shift and the refractive index shift has a linear relationship in a certain range. The relationship between the target DNA molecules at different concentrations and the reflectance spectrum red shift is shown in Figure 5.

Figure 5 shows both the QD-DNA red shift and the control DNA at different concentrations. When the concentrations of QD-DNA were 0.1 µM, 0.5 µM, 1 µM, 2.5 µM, and 5.0 µM, the reflectance spectra red shifts were 11 nm, 18 nm, 23.5 nm, 28 nm, and 31 nm, respectively. When the control DNA concentrations were 0.5 µM, 1 µM, 2.5 µM, and 5.0 µM, the reflectance spectra red shifts were 10 nm, 14 nm, 18 nm, and 23 nm, respectively. It can be seen from the figure that the QD-DNA red shift is larger than that of the control DNA at the same concentration. As shown in Figure 4, the red shift of the 0.1 µM QD-DNA at point a is equivalent to the red shift of the control DNA concentration at 0.5 µM (point a′). The red shift of the 0.5 uM QD-DNA at point b is similar to the red shift of the 2.5 µM control DNA (point b′). Finally, the red shift of the 1 µM QD-DNA at point c is a bit larger than that of the 5 µM control DNA concentration at point c′. That is to say, the detection sensitivity of QD-DNA is roughly five times that of conventional DNA detection. As can be seen from Figure 4, at DNA concentrations below 0.5 μM, the slope of the QD-DNA (14.34 nm/µM) data points is greater than the slope of controlled DNA (2.67 nm/µM) at the concentration range from 0.5 μM to 5 μM. Therefore, the QD-DNA detection sensitivity is at least five times higher than conventional DNA detection.

When QD-DNA is chosen as the target molecule for the reflectance spectrum shift, the sensitivity is improved due to the high refractive index of QDs. The QDs and DNA molecules are bonded together to increase the effective refractive index of DNA molecules, and then hybridized with a DNA probe in the PSM. This increases the PSM effective refractive index shift. To further improve the DNA detection sensitivity using QD as a marker, one should optimize the ratio of QDs to DNA. Table 1 shows the red shifts for two different ratios of QDs and DNA: 1:20 and 1:5 at different concentrations. The 1:20 ratio exhibits a 7 nm and a 12 nm red shift at 0.1 µM and 0.5 µM DNA concentrations, respectively. The red shift for the ratio of 1:5 is clearly larger than 1:20. However, the red shift in the 0.5 uM control DNA is 10 nm, so the detection sensitivity for 1:20 is not significantly improved. However, the detection sensitivity increased appreciably for the 1:5 ratio, indicating that the ratio of quantum dots and DNA has an impact on the detection sensitivity.

**Figure 6:**
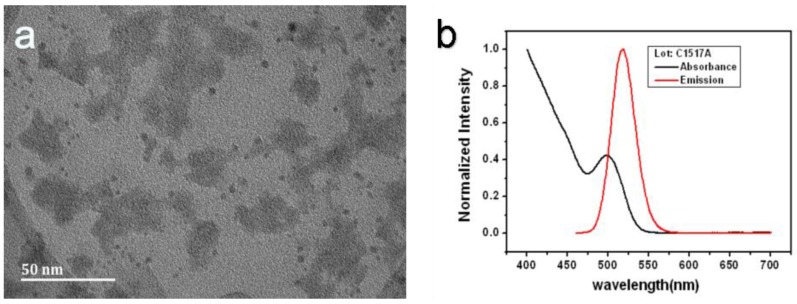
(**a**) The TEM image and (**b**) the absorption and emission spectra of QD.

The TEM image (Figure 6a) shows that the QD is about 4 nm, which is appropriate for our porous silicon holes. The refractive index of a QD is related to its size. Generally, the larger the QD is, the larger the refractive index. A QD with a large refractive index has a stronger spectral response, so the sensitivities scale with the QD size. However, the particle size will affect the particles entering into the porous silicon hole. The PSM structure detection sensitivity will be greatly reduced when the QDs cannot enter the pores, so the appropriate size is an important factor for increasing the sensitivity. The absorption spectroscopy image (Figure 6b) shows that the peak is located at 525 nm and the emission spectroscopy image (Figure 6b) shows that the peak is located at 500 nm. Our reflectance spectrum (Figure 4) shows that the dip is located at 650 nm; far away from the emission and the absorption peaks. Furthermore, the reflection light intensity is much stronger than the QD emission and absorption. Therefore, the QD absorption and emission have almost no impact on the sensing sensitivity.

At present, the detection limit of the latest porous silicon optical biosensor is about 40 nM [21,22]. In this paper, the sensor detection limit is also at this level when using the PSM to detect the control DNA without a quantum dot. However, the QD-labeled DNA detection sensitivity is five times higher than that of the conventional method. Using the QD-labeled DNA strand as an analyte has its own drawbacks; the method is more complicated and interferes with the target DNA more than the label-free detection method. As a result, the detection limit of this method is lower (0.1 nm/14.34 = 6.97 nM), and the sensitivity of this method is much higher.

## 4. Conclusions

In this work, we fabricated N-type macro-porous silicon microcavities and prepared QD-labeled DNA with carboxyl-CdSe/ZnS soluble QDs. By increasing the pore size, more quantum dots-labeled DNA molecules can be bonded to the microcavity pores. The effective refractive index is increased by combining the target DNA with the high-refractive-index QDs; thus, PSM spectral responses to the analytes are increased. The relationship between the QD-DNA concentration and the red shifts of the PSM reflectance spectrum was measured using a detection method based on refractive index changes. The results showed that the demonstrated method of QD-labeled DNA increased the reflectance spectrum red shift, improving sensitivity by a factor of five times compared to conventional DNA hybridization detection methods based on porous silicon. As a result, DNA labeled with QDs can be used for high sensitivity DNA sensing.

## Figures and Tables

**Table 1:** Red shifts for two different concentration ratios (1:20 and 1:5) of quantum dots and DNA at a DNA concentration of 0.1 µM and 0.5 µM. Results for control DNA are also given.

QD:DNA	1:20	1:5	ck DNA
0.1 μM	7 ± 0.8	11 ± 1	–
0.5 μM	12 ± 1	18 ± 1.5	10 ± 0.9
